# Compromise Effect in Food Consumer Choices in China: An Analysis on Pork Products

**DOI:** 10.3389/fpsyg.2020.01352

**Published:** 2020-06-30

**Authors:** Linhai Wu, Xiaoru Gong, Xiujuan Chen, Wuyang Hu

**Affiliations:** ^1^School of Business, Jiangnan University, Wuxi, China; ^2^Department of Agricultural, Environmental, and Development Economics, The Ohio State University, Columbus, OH, United States

**Keywords:** compromise effect, consumer preference, decoy information, pork products, anchoring effect

## Abstract

Compromise effect suggests that a product will have a higher chance to be chosen from a product choice set when its attributes are not the extremes (the best with the highest price or the worst with the lowest price). Few studies have examined compromise effect in food purchase. We investigate consumer pork purchase decision in the context of different decoy information intended to induce behavior and consider different presentation of decoy information. Furthermore, we explore compromise effect in relation to price, quality, and safety, which are directly related to consumer health. Results demonstrate that consumers exhibit significant compromise effects after receiving both low-price and high-price decoy information. However, when decoy information is presented after consumers have made choices without decoy information, their behavior changes systematically with a weakened compromise effect. This study highlights the implications of compromise effect in food marketing and policies related to food traceability and safety.

## Introduction

In the *Wealth of Nations*, Adam Smith proposed the concept of an “economic man” and rational human behavior. The core of this theoretical hypothesis considers people as subjects pursuing maximum personal utility (Richard, 2016). Based on this hypothesis, the rational choice theory argues that consumers attach utility to each option in a product choice set and will choose a product or group of products with maximized utility, regardless of the choice set in which the product is placed (Simonson and Tversky, 1992; Aleskerov and Monjardet, 2007). In recent years, psychology theories have also been used to study human economic behavior. According to Herbert A. Simon, a pioneer of modern decision theory, human choice is an adaptation mechanism with bounded rationality, rather than an optimum mechanism with complete rationality (Herbert, 1988). To a substantial degree, consumer choices depend on the environment in which the choices are made (Ahn and Vazquez Novoa, 2016). Therefore, under bounded rationality, consumers may not always choose the option that maximizes utility, and preference for a product depends on changes in consumption context (Huber et al., 1982; Payne et al., 1992; Li, 1995). This so-called context effect can be understood as a process in which consumers consider the absolute level of attributes of a target option, but at the same time are influenced by the location of the target option relative to other options in a choice set (Tversky and Simonson, 1993; Novemsky et al., 2007).

Huber et al. (1982) stated that compromise effect is a form of context effect. Simonson (1989) later summarized the concept of compromise effect, referring to it as a phenomenon “where an option is more likely to be chosen by consumers and attracts a larger portion of choices when it is a compromising or middle option in a choice set.” Simonson (1989) and Dhar and Maach (2004) argued that during the consumer decision-making process, the most likely context effect is compromise effect. Existing literature suggests that compromise effect influences consumer choices, leading to violations of the principle of utility maximization in traditional economic theory, and reflecting the characteristics of bounded rationality (Chen et al., 2018). Understanding compromise effects has proven useful for manufacturers in market positioning, brand promotion, and creation of competition strategies (Simonson and Tversky, 1992; Ran et al., 2004). For example, to help sell a high-priced product, marketers can introduce another option with a higher price compared to the target product as decoy information (Lichters et al., 2017). Although considerable research has focused on compromise effect in general consumer products, few have explored the effect in consumer food purchasing behavior. Food safety is of great importance to human health and has thus attracted considerable consumer attention, particularly in developing countries such as China. Therefore, in this paper, we use pork products as a case to explore the existence of compromise effect in food purchases and investigate the effect under different consumption contexts.

We chose pork because it is the most popular type of meat produced and consumed in China. Data from the United States Department of Agriculture (USDA) show that China’s pork production in 2017 was 53.50 million tons, accounting for 48.19% of the total pork output worldwide (111.03 million tons), with a per capita consumption of 39.12 kg, some 4.6 times the average of other countries. Nonetheless, pork and pork products have also triggered substantial quality and safety concerns in China. For example, Zhong and Wu (2019) reported that among the 22 436 quality and safety incidents regarding meat and meat products between 2006 and 2015, 65% of them were related to pork. These incidents were found in all areas of pork production, including breeding, slaughtering and processing, and circulation and sales, which accounted for about 39, 36, and 25% of the total number of pork-related incidents, respectively. Studying pork and measures to improve pork safety is thus relevant to food policy.

To enhance consumer knowledge and facilitate strengthened food safety, traceability measures of pork have received considerable attention in academia and industry (Heath and Chatterjee, 1995; Chuang and Yen, 2007; Chamorro et al., 2015). Although a complete food traceability system has not yet been established in China, many existing studies show that traceability plays one of the most important roles in improving consumer confidence in and consumption of pork products (Hobbs, 2004; Alfnes et al., 2018; Hou et al., 2019). As the increased costs of establishing a traceability system will also lead to higher sales prices of traceable pork, understanding consumer acceptance of traceable pork is crucial but is under-investigated in China. Therefore, in the present study, a separate goal is to also explore the future development of and consumer preference for traceable pork in China. With regard to compromise effect, if it exists in consumer purchase of traceable pork, food manufacturers and policy makers could take advantage of the compromise effect to improve acceptance and sales of traceable pork.

## Literature Review and Research Hypotheses

Previous studies have examined the consumer psychology aspects of compromise effect (Wernerfelt, 1995; Mourali et al., 2007). Dhar and Simonson (2003) proposed that consumers prefer the compromise option when they are uncertain but must make a choice so as to reduce losses associated with the extreme options and minimize expected loss. Simonson and Tversky (1992) and Sheng et al. (2005) called such consumer behavior the extreme circumvention principle. Consumer characteristics, product characteristics, and the external consumption environment are the main factors that influence compromise effect. However, consumer knowledge, psychological factors, product familiarity, consumption motivation, risk perception, and attitudes toward risks can all affect compromise effect (Mishra et al., 1993; Sheng et al., 2005; Mourali et al., 2007; Sinn et al., 2007; Drolet et al., 2009; Pinger et al., 2016). Although these factors are not the focus of this study, understanding these factors can also provide insight into the causes of compromise effect and should be a continued focus of future research.

For product characteristics, attribute importance (Belk, 1977), attribute comparability (Gourville and Soman, 2007), and brand effect of the option (Sinn et al., 2007) can affect compromise effect. In particular, brand image and reputation have a significant impact on consumer decision-making. Zeithaml (1988); Richardson et al. (1994), and González et al. (2015) demonstrated that consumers have higher expectation about product quality and higher willingness to buy when a product has a better brand image. Chuang and Yen (2007) studied the impact of the origin of various products (e.g., suitcases, watches, and sports shoes) on compromise effect and found that products from Germany can have more significant compromise effect on consumers than similar products from China. This is because a product originating from an origin with less quality image passes negative information about product quality to consumers and weakens the quality and price advantages of the compromise option.

Compromise effect is closely related to the external environment of consumption (Payne et al., 1992; Mourali et al., 2007). Kahneman and Tversky (1979) and Hoek et al. (2006) stated that when the same product information is presented differently, consumers may experience different information evaluation, psychological response, or attitude toward the product, and may consequently exhibit different preferences. Yoo et al. (2018) found that product information produces a different compromise effect on consumers when manufacturers present them in different formats. For example, compared to text, information expressed in numbers, figures, or symbols can have a greater impact on consumer preference and can produce a more significant compromise effect (Frederick and Lee, 2008; Kim, 2017). The same product information can also differ in its influence on compromise effect when presented in either positive or negative context comparing to other products. For instance, Schneider et al. (2001) found that physicians are more persuasive to patients when expressing the same information in a negative frame, whereas Levin and Gaeth (1988) found that consumers prefer the presentation of information in a positive frame. The same product information can also affect compromise effect when presented in a different order (Monk et al., 2016). Chen et al. (2011) revealed that compromise effect still occurs when consumers face decoy information presented to induce consumption, but when respondents are asked to first make a choice in the absence of decoy information and then to make a choice in the presence of decoy information, the results of the two choices differ significantly and compromise effect disappears. In our analysis, we also consider decoy information, which is defined as information given to consumers to induce their focus on certain product attributes instead of presenting new product attributes.

To summarize, extensive research has been conducted on compromise effect in consumer behavior. However, previous studies have primarily focused on compromise effect in consumer purchase of general products, with few studies exploring the effects on food purchase behavior. Thus, in the current study, we investigated compromise effect and its impact on marketing and consumer choice of food products. We proposed and tested the following five hypotheses using pork based on a consumer survey conducted in Wuxi, Jiangsu Province, China. We differentiated low-price and high-price decoy information, where the former was intended to induce consumers to consider low-priced products while the latter reversed the intention.

*H1_0_:* For consumers in a consumption context in the absence of decoy information, no compromise effect will occur in their behavior when choosing a pork product.

*H2_0_:* For consumers in a consumption context with low-price decoy information, no compromise effect will occur in their behavior when choosing a pork product.

*H3_0_:* For consumers in a consumption context with high-price decoy information, no compromise effect will occur in their behavior when choosing a pork product.

We further explored whether presenting decoy information at different stages of choice may affect choice behavior and compromise effect. This involved two stages: consumers were first asked to make a product choice without seeing any decoy information, and then they made another choice following the presentation of decoy information. Based on these considerations, we proposed the following two hypotheses:

*H4_0_:* Whether the low-price decoy information was presented to consumers after they have made a choice in absence of decoy information does not affect compromise effect in their behavior when choosing a pork product.

*H5_0_:* Whether the high-price decoy information was presented to consumers after they have made a choice in absence of decoy information does not affect compromise effect in their behavior when choosing a pork product.

## Experimental Design, Implementation, and Sample Characteristics

We chose pork hind leg meat since it is commonly consumed in China (Wang et al., 2011). Our preliminary survey indicated that pork hind leg meat is sold at a similar market price in different urban areas of Wuxi, Jiangsu Province, China, where our study was conducted. Studying consumer behavior regarding the same pork cut can effectively reduce the influence of non-experimental factors on research conclusions (Wang et al., 2011). We considered three pork characteristics directly related to food safety; i.e., traceable, Voted-Trusted-Brand (VTB), and origin-labeled.

Based on pork quality and safety risks in real markets and the Hazard Analysis Critical Control Point (HACCP) system in the pork supply chain in China, as well as the impact of information asymmetry on pork safety risks, a whole-process traceable pork information system should at least cover the three main aspects: breeding, slaughtering and processing, as well as circulation and sales.

Compared with conventional pork, traceable pork inevitably has higher production costs (Meuwissen et al., 2009). Traceability system covering more players in the supply chain can better help consumers identify and reduce pork quality risks, but at the same time can also increase the price of traceable pork (Bai et al., 2017; Matzembacher et al., 2018). At present, there is no complete list of prices for various traceable pork cuts on the Chinese market (Wu et al., 2015). Hence, since our survey was conducted in Wuxi, Jiangsu Province, consistent with Wu et al. (2018), and the time lag between our study and that of Wu et al. (2018) was relatively short, we used the same traceable pork prices set by Wu et al. (2018).

In the literature review, we described the relationship between brand and product origin and consumer behavior. We also investigated two other pork hind leg meat attributes: brand and origin-label, in addition to traceable pork. Due to inconsistent pork quality, since 2005, China has been vigorously developing the program known as the consumers’ Voted-Trusted-Brand (VTB) products. Each year, China’s Brand Name Association assesses and recommends pork brands as VTB. The assessment is based on market consumption for the year and consumer evaluation collected through the assocation’s country-wide consumer opinion surveys. Pork products sold in many food markets in China are considered safe but have only met safety standards at the minimum level. Compared with such pork products, VTB pork products have better perceived quality, more reliable safety, and usually higher prices.

In China, an origin-labeled product refers to a product that is from a specific geographical region, after which it is named and upon approval by the China National Accreditation Committee. This is consistent with the definition of country-of-origin-labeled products provided by the World Trade Organization in regard to intellectual property rights. Displaying the origin of a pork product in the form of a label can provide quality or safety information (Lim et al., 2014). Origin-label and traceability have different implications. The former establishes the overall product quality in association with the customs and culture of the certified geographical origin, whereas the latter identifies the specific enterprises or individuals involved in pork production and circulation.

We designed three pork products, represented by *x*, *y*, and *z* for each of the three types of pork hind leg (pork hind leg with each of the three safety and quality characteristics), as shown in Table 1. For each type of pork, we designed different decoy information. During the survey, all pork products were presented to participants in the order of *x*, *y* or *x*, *y*, *z* (no option *z* in some sample groups) to ensure consistency and comparability of the products. As stated previously, in addition to compromise effect, a second goal of our study is to specifically examine consumer behavior regarding traceable pork. Therefore, for traceable pork, we investigated whether consumer behavior demonstrated compromise effect under both high-price and low-price decoy information, whereas for the VTB and origin-labeled pork, we only implemented high-price decoy information due to article length constraints.

**TABLE 1:** Product options and decoy information for pork products.

Type of pork	Product option	Decoy information
Traceable pork hind leg meat	15.4 Yuan/500 g, traceable information covers breeding link (x). 16.8 Yuan/500 g, traceable information covers breeding and slaughtering links (y). 18.2 Yuan/500 g, traceable information covers breeding, slaughtering, processing, and sales links (z).	*Low-price decoy information*: Considering limited income and limited budget for food consumption, you can save much from buying inexpensive pork to purchase other necessary foods such as fruits and vegetables.
		*High-price decoy information*: There are risks in consuming ordinary pork. Long-term consumption of pork containing clenbuterol or veterinary drug residue is not conducive to health. In contrast, traceable pork has a relatively better guarantee of safety and quality.
Voted-trusted-brand (VTB) pork hind leg^ 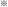 ^	24 Yuan/500 g, COFCO Joycome pork hind leg meat with skin (x) 36 Yuan/500 g, Zhili black pork hind leg meat (y) 40 Yuan/500 g, Netease Weiyang black pork hind leg meat (z)	*High-price decoy information*: There are certain quality and safety risks in consuming conventional pork, whereas VTB pork has generally better quality. A whole process safety traceability system is implemented for COFCO Joycome pork. Zhili black pigs are fed with pure grain, raised free-range, and have guaranteed quality. Netease Weiyang pork has not only assured quality but also a higher level of nutrition.
Origin-labeled pork hind leg^ 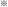 ^	40 Yuan/500 g, Guangxi Bama fragrant pork (x) 60 Yuan/500 g, Jinhua Liangtouwu pork hind leg meat (y) 80 Yuan/500 g, Daocheng Tibetan fragrant pork hind leg meat (z)	*High-price decoy information*: Origin-labeled pork is generally believed to have better quality and safety, in addition to its distinguished local characteristics. Guangxi Bama pork has a smooth taste, not greasy, and easy to digest. Jinhua Liangtouwu pork is popular country-wide for its thin skin, fine bones, not greasy meat, and rich flavor. Daocheng Tibetan pork is famous for its smooth, tender meat with high-level nutrition, good flavor, and leanness.

*^
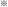
^Prices for traceable pork hind leg meat in Table 1 are based on Wu et al. (2018), and prices for VTB and origin-labeled pork were obtained from market surveys conducted in Wuxi, Jiangsu Province, China prior to this study.*

**FIGURE 1:**
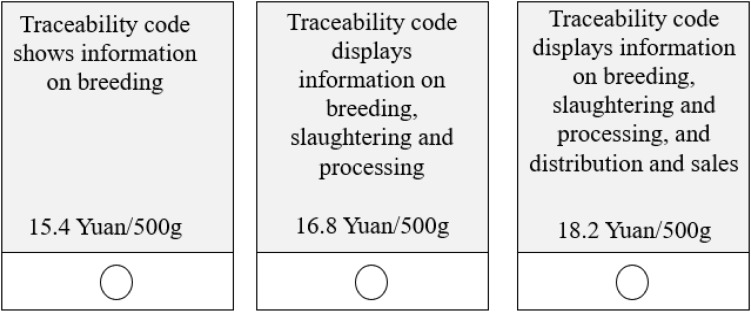
Sample of traceable pork choice set.

To present participants a choice context similar to a real consumption environment, we conducted our survey in large-scale super stores, pork shops, farmers’ markets, and shopping centers where consumers were engaging in actual grocery purchase in Wuxi, Jiangsu Province, China. The survey was conducted in August 2018 with a total of 1176 completed responses. Each respondent who completed the questionnaire was given a gift of 5 Yuan as compensation for their time. The interviewers were trained postgraduate students from a local university. The interviewers approached every third adult shopper came to their sight. Although respondents were not asked to make actual purchase, all pork products described in our survey were presented on site for participants to view and examine. Based on the objectives of the study, we designed eight sample groups, into which each survey participant was randomly assigned. Figure 1 shows a choice set of traceable pork. The options were represented by *x*, *y*, and *z* in the same left to right order in all sample groups. The number of pork products and number of choice sets each respondent review was different but the order of products presented in each choice set followed the order of *x*, *y*, and *z* in Table 1. The detailed description of the eight sample groups is as follows:

• Sample group #1 (no decoy information, two traceable pork options): Respondents made a choice in the absence of decoy information from a choice set composed two traceable pork hind leg meat products *x* and *y*, as shown in Table 1.
• Sample group #2 (no decoy information, three traceable pork options): Respondents made a choice in the absence of decoy information from a choice set composed of three traceable pork hind leg meat products *x*, *y*, and *z*, as shown in Table 1.
• Sample group #3 (low-price decoy information, two traceable pork options): Respondents were asked to first review the low-price information and then make a choice from a choice set composed of two traceable pork hind leg meat products *x* and *y*, as shown in Table 1.
• Sample group #4 (low-price decoy information, three traceable pork options): Respondents were asked to first review the low-price information and then make a choice from a choice set composed of three traceable pork hind leg meat products *x*, *y*, and *z* as shown in Table 1.
• Sample group #5 (first no decoy information, three traceable pork options, followed by low-price decoy information, same three traceable pork options): Respondents were first asked to make a choice in the absence of decoy information from a choice set composed of three traceable pork options as *x*, *y*, and *z* shown in Table 1. Then, low-price decoy information was presented and the same respondents were asked to make a choice from the same choice set.
• Sample group #6 (no decoy information, three types of pork products): Respondents made a choice in the absence of decoy information for each of the three types of products (traceable, VTB, and origin-labeled pork). For each type of product, three options were presented (i.e., products *x*, *y*, and *z* as shown in Table 1).
• Sample group #7 (high-price decoy information, three types of pork products): For each type of three pork products (traceable, VTB, and origin-labled pork), respondents were asked to first review the high-price information and then make a choice from a choice set composed of three options for each type of pork (i.e., products *x*, *y*, and *z* for each of the three types of pork hind leg meat, as shown in Table 1).
• Sample group #8 (first no decoy information, three types of pork products, followed by high-price decoy information, three types of pork products): For each of three types of pork (traceable, VTB, and origin-labeled pork), respondents were first asked to make a choice in the absence of decoy information from a choice set composed of three options (i.e., products *x*, *y*, and *z* for each of the three types of pork, as shown in Table 1). Then high-price decoy information was presented and the same respondents were asked to make a choice from each of the same choice set for the three types of products, respectively.

Characteristics of the total of 1176 adult consumers recruited into the study are shown in Table 2. These characteristics coincided with those in previous studies involving Chinese pork consumers (Wu et al., 2012).

**TABLE 2:** Descriptive statistics of respondent characteristics.

Statistical indicator	Category	Frequency	Effective percentage (%)
Gender	Male	540	45.92
	Female	636	54.08
Age (year)	18–25	552	46.94
	26–35	381	32.40
	36–45	117	9.95
	46–55	81	6.89
	56–65	39	3.31
	66–72	6	0.51
Family size	1	75	6.38
	2	180	15.31
	3	436	37.07
	4	246	20.92
	5	239	20.32
Level of education	Primary school and below	86	7.31
	Senior high school (including technical secondary school)	191	16.24
	College	255	21.68
	University	537	45.67
	Postgraduate and above	107	9.10
Annual household income	≤ 50000 Yuan	81	6.89
	50001–80000 Yuan	187	15.90
	80001–100000 Yuan	237	20.15
	100001–150000 Yuan	215	18.28
	>150000 Yuan	456	38.78

## Analysis Framework

Based on Chernev (2004), Eq. 1 calculates the change of the share of compromise option *y* being chosen between choice set {*x*, *y*} and {*x*, *y*, *z*}:

(1) $$\Delta \,p=P_{z}\,\left(y;x\right)-P\,\left(y;x\right)$$

where, *P*(y; x) is the share of option *y* relative to option *x* chosen from choice set {*x*, *y*}: and *P*_*Z*_(y; x) is the share of compromise option *y* relative to option *x* chosen from choice set {*x*, *y*, *z*}. *P*_*Z*_(y; x) indicates the attractiveness of compromise option *y* relative to *x* after the addition of the third traceable pork option *z* to the set, calculated using:

(2) $$P_{z}\,\left(y;x\right)=\frac{P\,\left(y;x,z\right)}{\left[P\,\left(y;x,z\right)+P\,\left(x;y,z\right)\right]}$$

where, *P*(y; x, z) is the share of compromise option *y* from choice set {*x*, *y*, *z*} and *P*(x; y, z) is the share of option *x* in choice set {*x*, *y*, *z*}.

*H1* can be tested by observing the share of option *y* chosen in sample groups #1 and #2. *P1 (y, x)* can be defined as the share of option *y* chosen in the two-option choice set {*x, y*} in sample group #1, and *P2_*z*_ (y, x)* can be defined as the share of option *y* chosen after option *z* was added to the set in sample group #2. If *P1 (y, x)* ≥ *P2*_*z*_ (*y*, *x*), then hypothesis *H1*_0_ cannot be rejected.

*H2* can be tested by observing the share of option *y* chosen in sample groups #3 and #4. If *P3 (y, x)* ≥ *P4_*z*_ (y, x)* (both with similar definitions as above), then hypothesis *H2*_0_ cannot be rejected.

*H3* can be tested by observing the share of options *y* and *z* chosen in sample groups #6 and #7. If *P7 (z, x)* ≥ *P7_*z*_ (y, x)*, then hypothesis *H3*_0_ cannot be rejected. In this design, tests were conducted for each of the three types of products.

Using sample groups #2 and #4, *P2_*z*_ (y, x)* and *P4_*z*_ (y, x)* can be obtained. The values for *P5a_*z*_ (y, x)* and *P5b_*z*_ (y, x)* (which represent the share of option *y* chosen without and with decoy information, respectively) can be obtained from sample group #5. Then *P4_*z*_ (y, x)* – *P2_*z*_ (y, x)* and *P5b_*z*_ (y, x)* – *P5a_*z*_ (y, x)* can be calculated, respectively. If *P4_*z*_ (y, x)* – *P2_*z*_ (y, x)* = *P5b_*z*_ (y, x)* – *P5a_*z*_ (y, x)*, then hypothesis *H4*_0_ cannot be rejected.

Using sample groups #6 and #7, *P6_*z*_(y, x)* and *P7_*z*_(y, x)* can be obtained. The values for *P8a_*z*_(y, x)* and *P8b_*z*_(y, x)* (with similar definitions as to *P5a_*z*_(y, x)* and *P5b_*z*_(y, x)*, respectively) can be obtained from sample group #8. Similarly, if *P7_*z*_(y, x) – P6_*z*_(y, x)* = *P8b_*z*_(y, x) – P8a_*z*_(y, x)*, hypothesis *H5*_0_ cannot be rejected. Tests can be conducted for each of the three types of pork products.

## Results and Discussion

**FIGURE 2:**
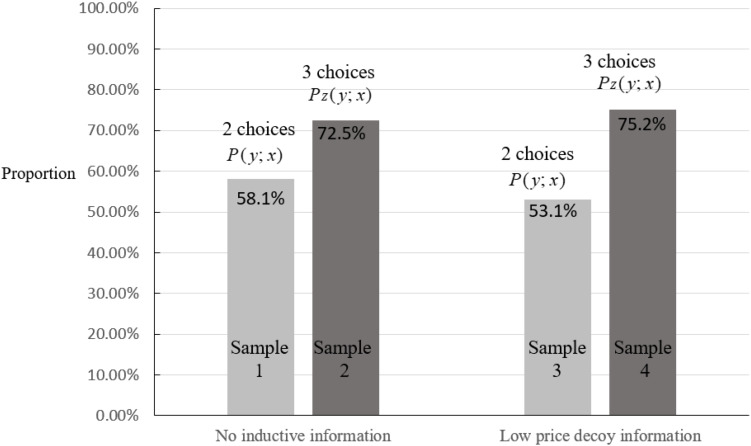
Choice of compromise option *y* under low-price decoy information.

In the absence of decoy information, the share of choosing compromise option *y* was 58.1% in sample group #1 (no decoy information and two traceable options) from choice set {*x, y*}, which increased to 72.5% in sample group #2 (no decoy information and three traceable options) from choice set {*x, y, z*} (see Table 3 and Figure 2), with ΔP = 21.94% (χ^2^ = 29.26, *p* < 0.001), *P1 (y, x) < P2_*z*_ (y, x).* Therefore, hypothesis *H1*_0_ can be rejected, indicating compromise effect occurring in consumer behavior in the absence of decoy information.

**TABLE 3:** Choices under low-price decoy information (%).

Product option	Sample group #2 (*N* = 149)	Sample group #4 (*N* = 149)	Sample group #5 (*N* = 147)		Chi-squared Test
	Without decoy information	With low-price decoy information	Without decoy information	With low-price decoy information	
15.4 Yuan/500 g, Traceability information covering breeding link (*x*)	18.12	14.77	23.49	48.64	
16.8 Yuan/500 g, Traceability information covering breeding and slaughtering links (*y*)	72.48	75.17	51.01	36.24	56.089***
18.2 Yuan/500 g, Traceability information covering breeding, slaughtering, and sales links (*z*)	9.40	10.06	25.50	15.12	

***Means significant at 1% significance level.

Under the low-price decoy information context, the share of choosing compromise option *y* was 53.1% in sample group #3 (low-price decoy information and two traceable options) from choice set {*x, y*}, which increased to 75.2% in sample group #4 (low-price decoy information and three traceable options) from choice set {*x, y, z*} (see Table 3 and Figure 2), with ΔP = 30.46% (χ^2^ = 45.35, *p* < 0.001), *P3 (y, x) < P4_*z*_ (y, x)*. Therefore, hypothesis *H2*_0_ is rejected, showing compromise effect occurring in consumer behavior under low-price decoy information context.

We also test the influence of high-price decoy information on participant choices, as presented in Table 4. In group #7 (high-price decoy information for all three types of pork), the share of compromise option *y* chosen by participants from the three traceable pork choice set {*x*, *y*, *z*} was 45.19% and the share of option *z* was 39.95% (χ^2^ = 23.08, *p* < 0.05), suggesting that option *y* shows a compromise effect under this context. Also in group #7, the share of the VTB pork option *y* chosen by participants was 54.73% and that of option *z* was 14.46% (χ^2^ = 36.36, *p* < 0.05), demonstrating the existence of compromise effect in the share of option *y*. The share of origin-labeled pork option *y* chosen by group #7 was 41.76%, and that of option *z* was 31.49% (χ^2^ = 5.20, *p* < 0.01), suggesting the existence of compromise effect in option *y*. Based on these results, *H3*_0_ can be rejected and participant choice behavior showed a compromise effect. As shown in Table 3, *P2_*z*_ (y, x)* was 72.48% and *P4_*z*_ (y, x)* was 75.17%. For sample group #5 (first no decoy information and then low-price decoy information for all three types of pork), the values for *P5a_*z*_ (y, x)* and *P5b_*z*_ (y, x)* were 51.01 and 36.24%, respectively. Hence, *P4_*z*_ (y, x)–P2_*z*_ (y, x)*≠ *P5b_*z*_ (y, x)–P5a_*z*_ (y, x)* (χ^2^ = 56.089, *p* < 0.01). Therefore, hypothesis *H4*_0_ can be rejected. In other words, whether the low-price decoy information is given after consumers have experienced non-decoy information affected compromise effect.

**TABLE 4:** Choices under high-price decoy information (%).

Information presentation	Product option	Traceable pork	Voted-trusted- Brand (VTB) pork	Origin-labeled pork
Sample group #6, without decoy information (*N* = 144)	x	22.92	37.22	37.27
	y	47.50	45.14	40.92
	z	29.58	17.64	21.81
Sample group #7, with high-price decoy information (*N* = 148)	x	14.86	30.81	26.75
	y	45.19	54.73	41.76
	z	39.95	14.46	31.49
Sample group #8, without decoy information, then with high-price decoy information (*N* = 144)	x	20.09	32.62	34.76
	y	41.67	48.80	45.88
	z	38.24	18.58	19.36
	x	17.36	24.81	22.78
	y	39.59	30.69	32.64
	z	43.05	44.50	44.58
Chi-squared test		2.295	20.272***	2.261

***Means significant at 1% significance level.

We next tested hypothesis *H5*, based on findings shown in Table 4. Results showed that *P7_*z*_(y, x)*, *P8a_*z*_(y, x)*, and *P8b_*z*_(y, x)* for traceable pork compromise option were 45.19, 41.67, and 39.59%, respectively, hence, *P7_*z*_(y, x)–P6_*z*_(y, x)*≠ *P8b_*z*_(y, x)–P8a_*z*_(y, x) (χ^2^* = *2.295, p* = *0.317)*. Values of *P7_*z*_(y, x)*, *P8a_*z*_(y, x)*, and *P8b_*z*_(y, x)* for VTB pork compromise option were 54.73, 48.80, and 30.69%, respectively, hence, *P7_*z*_(y, x)–P6_*z*_(y, x)*≠ *P8b_*z*_(y, x)–P8a_*z*_(y, x) (χ^2^* = *20.272, p* < *0.01)*. Values of *P7_*z*_(y, x)*, *P8a_*z*_(y, x)*, and *P8b_*z*_(y, x)* for the origin-labeled pork compromise option were 41.76, 45.88, and 32.64%, respectively, hence *P7_*z*_(y, x)–P6_*z*_(y,x)*≠ *P8b_*z*_(y, x)–P8a_*z*_(y, x)* (*χ^2^* = *2.261*, *p* = *0.3230*). Therefore, *H5*_0_ can be rejected for all three types of pork products. In other words, whether the high-price decoy information is given after consumers have experienced non-decoy information affected compromise effect.

Chen et al. (2011) revealed that the likelihood of a compromise option being chosen by participants decreases under decoy information in comparison to when no decoy information is presented. In contrast, however, our results showed that participants still preferred pork product option *y* and showed a significant compromise effect.

Results of testing hypothesis *H4* based on Table 4 shows participant choices under different consumption contexts in the absence of decoy information and in the presence of low-price decoy information. When participants made choices without decoy information (sample group #2). Under this context, compromise option *y* was selected most often, and a significant compromise effect occurred. In sample group #4, participants made a choice in the presence of decoy information. Comparing sample groups #2 to #4, compromise option *y* exhibited an absolute advantage and had the largest choice share. However, in sample group #5, we found that if respondents first made a choice in the absence of decoy information and then made a choice again after receiving low-price decoy information, the share of option *y* decreased from 51.01 to 36.24% (*χ*^2^ = 56.089, *p* < 0.01) and compromise effect disappeared. Further analysis of sample groups #4 and #5 after the addition of low-price decoy information showed that consumer choices differed greatly. Specifically, the share of option *x* chosen by participants increased from 14.77% in sample group #4 to 48.64% in sample group #5, whereas that of option *y* decreased from 75.17 to 36.24%, respectively. Option *x* was traceable pork with the lowest price. The intention of the decoy information was to induce participants to consider low-priced traceable pork. However, this decoy effect did not occur in sample group #4.

Similar results were observed when testing *H5* based on Table 3. We observed the three choice decisions made by participants in sample group #8 and found that without decoy information, the shares of compromise option *y* were 41.67, 48.80, and 45.88%, respectively. When the same participants chose again after the addition of high-price decoy information, the shares of compromise option *y* decreased to 39.59, 30.69, and 32.64%, respectively. Further analysis revealed that the share of option *y* chosen by sample group #8 under a high-price decoy information context was significantly lower than that of option *z*, which then became the most preferred product.

These findings suggested that a change in the presentation of decoy information had an influence on compromise effect. Presenting the decoy information after a choice without decoy information weakened compromise effect (in sample group #5 and #8). However, for sample groups #4 or #7, significant decoy effect was still observed, even though these consumers were presented with the same decoy information. Overall, we find that when facing three pork options in a choice set {*x*, *y*, *z*}, participants generally considered it attractive to choose the compromise option, thus showing a clear compromise effect. When the presentation of decoy information is moved after a choice without decoy information, participant preference changed to exhibit less compromise effect.

## Conclusion and Implications

This paper focused on understanding compromise effect in consumer choices of pork products under a consistent sequence of product presentation. We also examined the impact of different decoy information and whether the information was presented with or without the respondents first making a choice with no decoy information. As demonstrated, consumer decision-making in pork purchases showed significant compromise effect. Furthermore, compromise effect exists under decoy information featured as high-price and low-price information in this research. However, when consumers made an initial choice without any decoy information, and then chose again following the presentation of decoy information, their choices were more spread out across all products in the choice set and the compromise effect disappeared. This is a reflection of how changes in the presentation of decoy information influenced consumer behavior.

In this study, we used pork to demonstrate that consumer choices exhibited compromise effect with or without decoy information. However, the size of compromise effect may not be identical for all types of food. As the main source of animal protein for Chinese consumers and a basic component of the Chinese CPI, demand price elasticity for pork is lower than that for most other foods. If a product with even lower demand elasticity was used in this study (for instance, rice or wheat flour), the intensity of compromise effect may need reevaluation when these products are compared in one choice set with those having higher demand elasticity. However, within one product category, we expect compromise effect still to take place.

In sample groups #5 and #8 in our study, in order to ensure that the choices of the same respondents before and after the decoy information were not influenced by the order of product presentation, we maintained a consistent product order in the choice set before and after the presentation of decoy information. This also allows a more direct comparison with choices made in other sample groups. This means that during our entire study, the products were always presented to the respondents in the order of *x*, *y*, and *z*. A drawback of this approach is that the order effect may be confounded with compromise effect. Furthermore, compromise effect is being tested predominantly in the literature by varying the number of products/attributes in a choice set. Another layer of confounding may occur between the number of products/attributes and compromise effect, although we argue that based on our consistent discovery of compromise effect in product choice sets with different numbers of products/attributes, the possible confounding effect may not significantly undermine our findings. Limited by the current length of the article, we have not specifically tackled these potential confounding effects, which may be a valuable subject for exploration in future research.

The conclusions of this study have several policy implications. Much of our findings on traceable pork suggest that the Chinese government should encourage manufacturers to produce traceable pork with diverse levels of traceable information at varied prices to form a traceable pork system. This will not only satisfy the diverse demand for traceable pork, if traceability is deemed as the prominent tool to assist the construction of a safer national pork supply chain, manufacturers should be encouraged to increase the market share of traceable pork products by harnessing the compromise effect to promote traceable pork.

## Data Availability Statement

The datasets analyzed in this manuscript are not publicly available to protect subjects’ privacy, and to comply with regulations set by Jiangsu Social Science Fund Major Project. Requests to access the datasets should be directed to the corresponding author.

## Ethics Statement

The studies involving human participants were reviewed and approved by human ethics review board of the Jiangnan University of China. Written informed consent from the participants was not required to participate in this study in accordance with the national legislation and the institutional requirements.

## Author Contributions

LW: proposing the research direction of the thesis and designing the structure of the article. XG: questionnaire and manuscript drafting. XC and WH: revise and propose.

## Conflict of Interest

The authors declare that the research was conducted in the absence of any commercial or financial relationships that could be construed as a potential conflict of interest.
